# Metabolic engineering and transcriptomic analysis of *Saccharomyces cerevisiae* producing *p*-coumaric acid from xylose

**DOI:** 10.1186/s12934-019-1244-4

**Published:** 2019-11-05

**Authors:** Gheorghe M. Borja, Angelica Rodriguez, Kate Campbell, Irina Borodina, Yun Chen, Jens Nielsen

**Affiliations:** 1 The Novo Nordisk Foundation Center for Biosustainability, Technical University of Denmark, 2800 Lyngby, Denmark; 2 Department of Biology and Biological Engineering, Chalmers University of Technology, 412 96 Gothenburg, Sweden; 3 BioInnovation Institute, Ole Måløes Vej 3, 2200 Copenhagen N, Denmark; 4 Present Address: The Bioinformatics Centre, Section for Computational and RNA Biology, Department of Biology, Faculty of Science, University of Copenhagen, Ole Maaloes Vej 5, 2200 Copenhagen, Denmark

**Keywords:** *Saccharomyces cerevisiae*, Transcriptome, *p*-Coumaric acid, Xylose, RNA*seq*

## Abstract

**Background:**

Aromatic amino acids and their derivatives are valuable chemicals and are precursors for different industrially compounds. *p*-Coumaric acid is the main building block for complex secondary metabolites in commercial demand, such as flavonoids and polyphenols. Industrial scale production of this compound from yeast however remains challenging.

**Results:**

Using metabolic engineering and a systems biology approach, we developed a *Saccharomyces cerevisiae* platform strain able to produce 242 mg/L of *p*-coumaric acid from xylose. The same strain produced only 5.35 mg/L when cultivated with glucose as carbon source. To characterise this platform strain further, transcriptomic analysis was performed, comparing this strain’s growth on xylose and glucose, revealing a strong up-regulation of the glyoxylate pathway alongside increased cell wall biosynthesis and unexpectedly a decrease in aromatic amino acid gene expression when xylose was used as carbon source.

**Conclusions:**

The resulting *S. cerevisiae* strain represents a promising platform host for future production of *p*-coumaric using xylose as a carbon source.

## Background

Aromatic amino acids (AAAs) and their derivatives (AAADs) are valuable chemicals, that also serve as precursors for a variety of other industrially relevant compounds. Currently these compounds have a global market of ~ $8 billion, which is projected to reach ~ $20 billion by 2020 [1, 2]. The majority of AAADs (see Table 1), such as flavonoids and polyphenols, are produced from either non-renewable fossil fuels or by plant extraction, which suffers from either non-sustainable production or low yields. To reduce the dependency on fossil fuels for this process, whilst meeting the growing demand for AAADs, renewable sources of biomass have subsequently been explored as substrate for AAAD production.

**Table 1:** Examples of microbial production of aromatic amino acid derivatives (AAAD)

Product	Titer	Yield	Organism	Feedstock	Culture style	Reference
*trans*-Cinnamic acid	151 mg/L	0.016 g/g	*E. coli*	Arabinose	Batch (shake flask)	[68]
Styrene	29 mg/L	1.44 mg/g	*S. lividans*	Glucose	Batch (shake flask)	[69]
*p*-Hydroxystyrene	2.52 g/L	0.027 g/g	*P. putida*	Glucose	Fed-batch	[70]
*p*-Hydroxybenzoic acid	2.3 g/L	0.11 g/g	*E. coli*	Glucose, xylose	Fed-batch	[71]
*p*-Aminobenzoic acid	215 mg/L	N.E.	*S. cerevisiae*	Glycerol, ethanol	Fed-batch	[72]
*p*-Coumaric acid	2.4 g/L	0.013 g/g	*S. cerevisiae*	Glucose	Batch	[12]
Resveratrol	0.8 g/L	NE	*S. cerevisiae*	Glucose, ethanol	Fed-batch	[35]

*NE* not estimated

So far, the biotechnology industry has investigated use of prokaryotes for AAAD production, for example, in modified strains of *Escherichia coli* [3–5], *Pseudomonas putida* [6], and *Corynebacterium glutamicum* [7, 8], with glucose being used as the primary carbon source. Exploratory studies in plants have also proven it to be possible to produce a diverse array of AAADs in tobacco [9], tomato [10] and *Arabidopsis thaliana* [11]. To date however, the highest titers of 2.4 g/L of *p*-coumaric acid have been achieved using microbial fermentation with the yeast *Saccharomyces cerevisiae* [12]. This yeast is therefore considered a promising cell factory platform for AAAD production, in particular due to its ability to express functional P450 proteins that are required for synthesising many AAADs, a feat that can otherwise be challenging when prokaryotes are used. This is well illustrated by the implementation of a commercial production of resveratrol by an engineered yeast strain, a process that has been established by the biotech company Fluxome AS and later acquired by Evolva AG.

*Saccharomyces cerevisiae* has proven a robust cell factory platform for diverse industrial applications, being able to produce food and beverage supplements [13, 14], rotavirus-like-particles [15], antibodies [16], therapeutic proteins [17], sesquiterpenes [18], isoprenoids [19], succinic acid [20], amongst other industrially relevant chemicals. Extensive tools have also been developed, such as CRISPR-Cas gene editing, for rapid and effective genetic manipulation of this yeast [21–23]. Moreover, it has proven possible to reconfigure *S. cerevisiae*’s metabolism using evolutionary engineering such as adaptive laboratory evolution [24]. With this approach, yeast is genetically adapted in response to adverse conditions based on the principles of natural selection. Using deep sequencing and reverse engineering, causal mutations can then be introduced into a parent strain, to confer its resistance to stress conditions such as high temperatures [25], osmotic stress [26], low pH [27], or toxic products [28]. Taken together, these attributes make *S. cerevisiae* a promising candidate for use as a platform strain for the production of AAADs [29–31].

Previous studies that have used *S. cerevisiae* for the production of AAA and AAADs have been performed by eliminating feedback control at critical points in the shikimate pathway, which is responsible for the synthesis of phenylalanine, tyrosine and tryptophan. This approach included using a mutated version of chorismate mutase, *ARO7,* and 3-deoxy-d-arabinoheptulosonate 7-phosphate (DAHP) synthase, *ARO4*, which enabled a 200-fold increase in AAAD compound production compared to the reference strain [32]. Many others AAADs such as resveratrol [33], naringenin [34], pinostilbene and pterostilbene [35] have also been successfully produced when glucose acts as the main carbon source. One valuable AAAD that belongs to a group of phenolic compounds commonly found in the plant kingdom is *p*-coumaric acid. This compound has been known to confer several health promoting physiological effects, such as anti-anxiety, anti-cancer, anti-oxidant, anti-inflammatory and anti-microbial activity [36]. *p*-Coumaric acid is also extensively employed respectively in the cosmetic, pharmaceutical and food industry, acting as a building block for more complex compounds such as noscapine [37]. Despite *p*-coumaric acid’s wide-ranging uses, an economically viable and sustainable workflow for its production at industrial scale is lacking, with raw materials typically being an important point to consider in terms of process cost and design.

Here, yeast fermentations predominantly use 2% glucose as the carbon source, wherein the Crabtree effect, also known as overflow metabolism, is in effect. This phenomenon subsequently leads to the extracellular accumulation of energetically expensive metabolites during fermentation, such as acetate, ethanol, and glycerol, with decreased carbon allocation for the desired value-added end product [38, 39]. One solution to avoid the Crabtree effect and redirect carbon flux towards biomass and AAAD production is by using non-fermentable sugars such as pentoses like xylose. After glucose, xylose is the second most abundant sugar in the world [40]. Subsequently, over the last 10 years, considerable efforts have been made by various research groups to transform xylose into a substrate for *S. cerevisiae,* thereby increasing its substrate range capability [40–42]. For example, Scalcinati et al. [43] developed a xylose utilizing strain (CMB.GS010) through adaptive evolution, which consumes xylose as the sole carbon source via the expression of PsXYL1 (xylose reductase, XR), PsXYL2 (xylitol dehydrogenase, XDH) and PsXYL3 (xylulose kinase, XK) from *Pichia stipitis*. The resulting strain had reduced overflow metabolism, with maximized carbon flux towards biomass production, and a biomass yield (Cmol Cmol^−1^) of approximately four times that of glucose [43].

Here, using metabolic engineering and a systems biology approach, we generated a platform strain in *S. cerevisiae* (CMB.GS010) that utilises xylose as the sole carbon source for *p*-coumaric acid production. The resulting strain achieved a final *p*-coumaric acid titer of 242 mg/L, representing a 45-fold-increase over our condition with glucose. Furthermore, we used transcriptomic analysis to characterize the resulting strain under aerobic and controlled (carbon limited) fermentation conditions to determine how metabolism was altered when cells are grown on xylose instead of glucose.

## Results

### Engineering a xylose utilization strain for the production of *p*-coumaric acid

To investigate the physiological impact of the two carbon sources, glucose and xylose, and specifically how xylose affects *p*-coumaric acid production, we used the strain CMB.GS010 that had already been evolved to grow on xylose in a previous study [43]. The phenotype reported by Rodriguez et al. [44] resulted in a higher *p*-coumaric acid producer strain. Therefore, the genetic modifications of the best *p*-coumaric acid producer were performed in CMB.GS010 as a background strain. Specifically, to increase *p*-coumaric acid production, we first reduced by-product formation by knocking out both *ARO10* and *PDC5*. Then we expressed shikimate kinase II (*aroL*) from *E. coli* and tyrosine ammonia-lyase (*TAL*) from *Flavobacterium johnsoniae*, with tyrosine deamination enabling the production of *p*-coumaric acid [45]. Finally, to increase the overall carbon flux through the aromatic amino acid pathways, we overexpressed feedback-resistant versions of DAHP synthase and chorismate mutase producing the final strain used for characterisation, ST4274.

### Physiological characterization of *p*-coumaric acid producing strain under controlled conditions

**Fig. 1:**
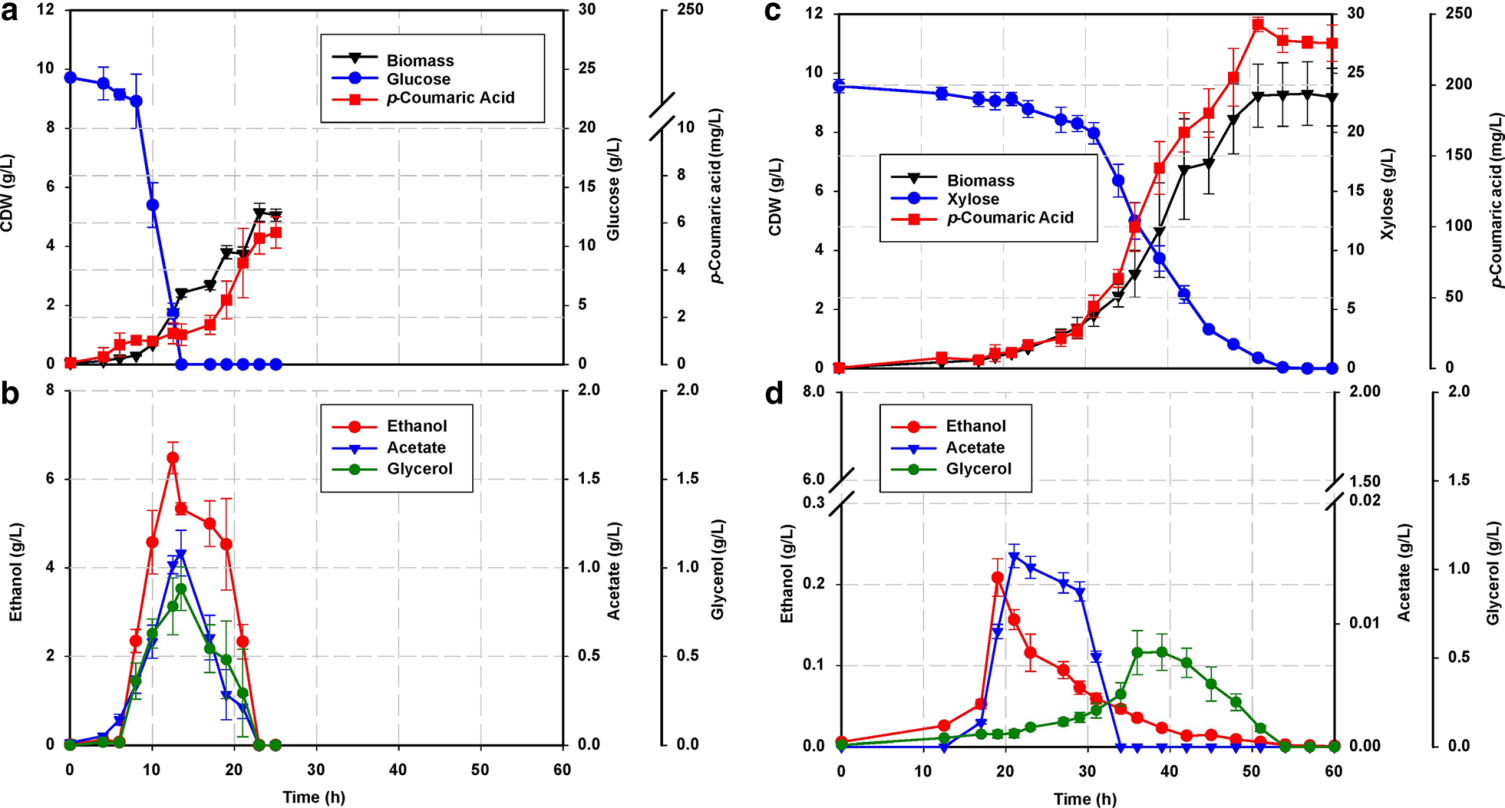
Microbial production of *p*-coumaric acid in strain ST4274 during growth on: **a** glucose and **b** xylose in batch cultivations with an initial substrate concentration of 25 g/L. Top panels: substrate, biomass and *p*-coumaric acid. Bottom panels **c**, **d** ethanol, acetate and glycerol concentrations (n = 4 ± sd)

ST4274 was evaluated in small-scale bioreactors under well-defined, aerobic conditions, with two sets of batch cultivations carried out independently, containing 25 g/L glucose and 25 g/L xylose respectively (Fig. 1). Under our conditions, when glucose was used as the sole carbon source, a maximum concentration of 1.8 ± 0.02 mg/L of *p*-coumaric was produced. And following glucose exhaustion, and a second (respiratory) growth phase (14–24 h) wherein the remaining organic acids were consumed in conjunction with the re-assimilation of ethanol, acetate, and glycerol, (produced by the cell during the glucose consumption phase), *p*-coumaric acid titer increased to 5.35 ± 0.32 mg/L. These values are notably less than previously reported, something which could be attributed to inter-strain differences between studies. Nonetheless, overall these results indicate that *p*-coumaric acid production occurs at higher levels during respiratory metabolism relative to fermentative metabolism.

To demonstrate that *p*-coumaric acid can be produced from xylose alone, a second set of cultivations were performed. Under these conditions, a maximum concentration of 242 ± 5 mg/L *p*-coumaric acid was produced after 60 h of cultivation (Fig. 1b). This represents a 45-fold increase compared with glucose conditions. During growth on xylose (Fig. 1b), it is also clear that only one growth phase occurs, similar to the growth profile of respiring cells and contrasting to cells grown on glucose that undergo fermentation followed by respiration after the diauxic shift. During growth on xylose, the secretion of organic acids was also observed to be ca. tenfold lower than during growth on glucose, reflecting a decreased effect of overflow metabolism. However, the maximum specific growth rate on xylose was also threefold lower (Table 2), similarly, the specific xylose uptake rate was ca. tenfold lower than the glucose uptake rate, indicating an overall decrease in carbon uptake and a concomitant reduction in biomass formation when xylose was used as the sole carbon source. Taken together, these results suggest that cells prefer glucose as a carbon source relative to xylose, however, as the titer of *p*-coumaric acid was 45-fold higher under xylose conditions, despite the lower growth rate, xylose remained the optimal carbon source for *p*-coumaric acid production. This higher *p*-coumaric acid titer could be attributed to cells undergoing a respiratory like metabolism, wherein overflow metabolism does not occur, allowing for carbon flux to be utilised more efficiently for *p*-coumaric acid synthesis. This observation matches results seen for the second growth phase on glucose, as cells utilise ethanol following the post-diauxic shift, where activation of respiratory metabolism occurred alongside an increase in *p*-coumaric acid production (Table 3).

**Table 2:** Physiological parameters for batch and chemostat cultivation

	Substrate	
	Glucose	Xylose
Batch		
µ_max_ (h ^−1^)	0.32 ± 0.02	0.11 ± 0.01
Biomass concentration (Cx in g_DW_/L)	4.5 ± 0.8	12.16 ± 0.9
*p*-coumaric acid (mg/L)	5.35 ± 0.32	242 ± 12
Y_SX_ (g/gDW)	0.13 ± 0.02	0.5 ± 0.08
Y_PX_ (mg/gDW)	1.18 ± 0.12	19.90 ± 2
Y_PS_ (mg/gDW)	0.214 ± 0.02	9.68 ± 0.59
*q*_*s*_ (g gDW/h)	2.46 ± 0.43	0.23 ± 0.02
*q*_*gly*_ (g/gDW h)	0.10 ± 0.02	0.001 ± 3 × 10^−4^
*q*_*eth*_ (g/gDW h)	1.37 ± 0.38	0.002 ± 1 × 10^−4^
*q*_*ac*_ (g/gDW h)	0.1 ± 0.02	1 × 10^−4^ ± 1 × 10^−5^
*q*_*p*_ (g/gDW h)	0.38 ± 0.02	2.19 ± 0.31
Q_*p*_ (g/gDW/h)	0.22 ± 0.02	4.75 ± 0.45
*q*_*O2*_ (mmol/C-mmol/h)	11.1 ± 0.6	0.96 ± 0.07
*q*_*CO2*_ (mmolCO_2_/C-mmol/h)	15.02 ± 0.78	1.06 ± 0.09
RQ (–)	0.70	1.11
Chemostat		
D (h^−1^)	0.048 ± 0.003	0.047 ± 0.002
Feeding solution (Cx in g/L)	7.5	15
Biomass concentration (Cx in g_DW_/L)	2.87 ± 0.3	3.62 ± 0.3
Residual substrate (g/L)	ND	7.66 ± 0.3
*p*-coumaric acid (mg/L)	2.23 ± 0.04	55.5 ± 3
*q*_*s*_ (mmol/gDW/h)	1.41 ± 0.09	0.55 ± 0.02
*q*_*gly*_ (mmol/gDW/h)	2.54E^−03^	ND
*q*_*eth*_ (mmol/gDW/h)	1.70E^−01^	ND
*q*_*ac*_ (mmol/gDW/h)	2.84E^−03^	ND
*q*_*O2*_ (mmol/gDW/h)	0.25 ± 0.02	0.11 ± 0.02
*q*_*CO2*_ (mmolCO2/gDW/h)	0.22 ± 0.22	0.09 ± 0.02
RQ (–)	1.13 ± 0.03	1.22 ± 0.03
Dissolved oxygen (%)	> 80	> 80

Data are means from four independent fermentations (n = 4 ± standard deviation, sd)

*RQ* respiratory quotient, *ND* not detected

### Transcriptomic analysis

**Fig. 2:**
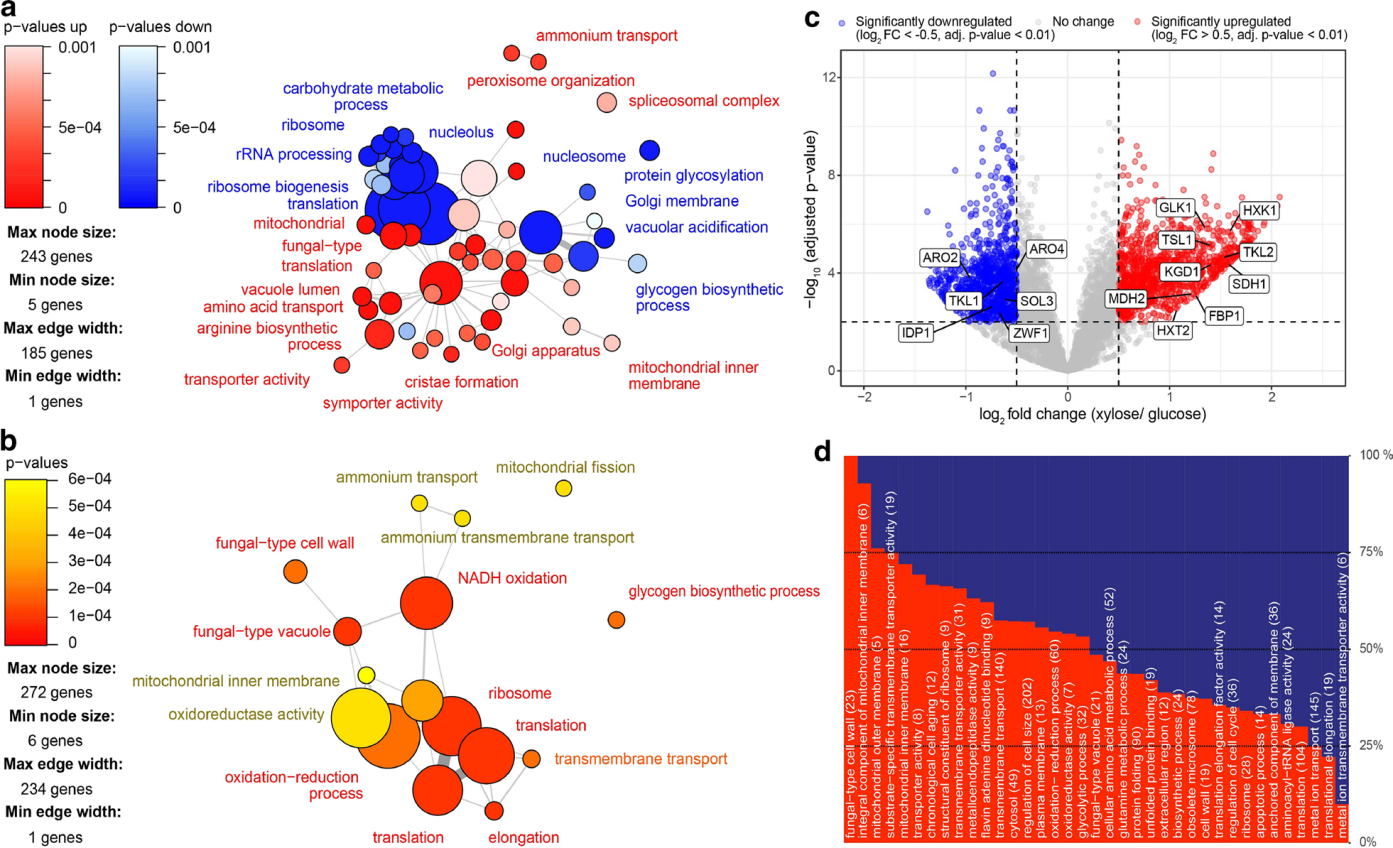
Gene-set analysis (GSA) during glucose and xylose carbon limitation in chemostat cultures. Top panels **a**, **b** Gene sets were defined by gene ontology (GO) terms, which show significant (p < 0.001) differential expression when grown on xylose compared to glucose. **c** The transcriptome profile under carbon limitation. X-axis specifies the log_2_ fold-change (log_2_FC) xylose/glucose, y-axis specifies the negative logarithm to the base 10 p-values. Red and blue points reflect the filtering criteria (log_2_FC > 0.5 and < − 0.5, and adjusted p-value < 0.01). Grey dots represent genes without significant expression. Examples of several genes, which were significantly up or down regulated under xylose conditions are indicated and commented on further in the text. **d** Significantly (p < 0.01) enriched GO terms are shown in terms of percentage of genes either up- or downregulated under carbon limitation. The top upregulated GO terms are shown in red while the lower downregulated GO terms are shown in blue

To understand more clearly how cells respond to growth on xylose whilst producing *p*-coumaric acid, we sampled from the chemostat cultures and quantified the cell’s transcriptional response to both carbon sources respectively, by performing RNA sequencing, differential expression and gene-set analysis (Fig. 2). Under xylose growth, upregulated GO-terms included those involved in transport activities, biosynthetic processes and membrane functions (Fig. 2a, d). GO-terms enriched in downregulated transcripts were related to carbohydrate metabolic processes and protein translation (Fig. 2a, d) with transcripts related to fungal cell wall, oxidation–reduction processes and ammonium transport being significiantly differentially expressed when directionality of regulation was not considered (Fig. 2b). These results would suggest cells are adapting to xylose by optimising their ability to take up extracellular xylose and synthesise biomass despite this carbon being suboptimal for the cell, relative to growth on glucose as well as there being a poor transport affinity for this pentose sugar. The downregulation of protein translation also corroborates the observation of the slower growth rate on xylose, as cells here may be re-allocating protein to optimise metabolism as opposed to growing quickly. The changes in oxidation and reduction processes also reflects cells undergoing respiratory metabolism as opposed to fermentative metabolism, as is the case when glucose is used [43].

**Fig. 3:**
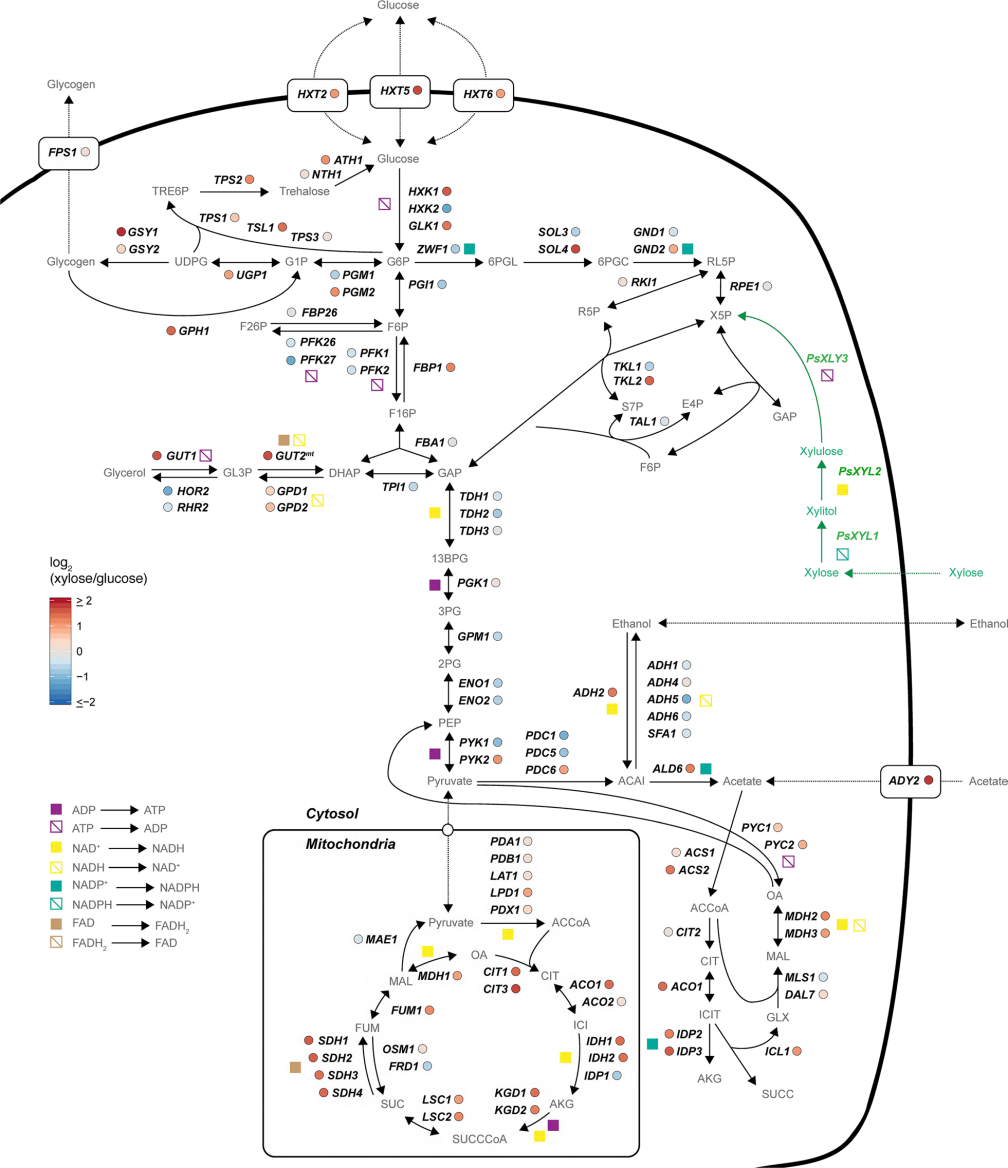
Gene expression levels of central carbon metabolic pathways. Tricarboxylic acid (TCA) cycle, glyoxylate pathway, gluconeogenesis, glycogenesis and pentose phosphate pathway (PPP) are presented. The comparative analysis includes the log_2_ fold-change (log_2_FC) xylose/glucose under carbon limitation conditions. The green label indicates overexpressed enzymes, “fbr” indicates feedback-resistant

To determine the role of central carbon metabolism, which generates the main building blocks for *p*-coumaric acid biosynthesis, these pathways specifically were examined further (Fig. 3). Here, significantly up-regulated transcripts were involved in respiration, in the tricarboxylic acid (TCA) cycle and glyoxylate pathway, correlating well with the observations found in the physiological data, which show an active respiratory system (Table 2). For example, in the glyoxylate pathway, *ICL1* encoding isocitrate lyase, *MLS1* and *DAL7* encoding two malate synthases, and *MDH2*, *MDH3* encoding two malate dehydrogenases were significantly up-regulated when ST4274 was grown on xylose. Additionally, the glyoxylate pathway had a similar up-regulation. This included succinate dehydrogenase, α-ketoglutarate dehydrogenase and succinyl-CoA ligase *SDH1*, *SDH2*, *SDH3*, *SDH4*, *KGD1*, *KGD2* and *LSC2*, suggesting that the glyoxylate shunt is up-regulated during respiratory metabolism, which has also been shown previously [46]. We also found, two isoenzymes of isocitrate dehydrogenase *IDP1*, fumarate reductase *FRD1* and mitochondrial malic enzyme *MAE1* to be significantly down-regulated in xylose limited conditions (Fig. 3a), supporting the hypothesis that the activity of the glyoxylate shunt is higher. This would suggest that cells adapt to growth on xylose by activating respiratory metabolism, and specifically the TCA cycle, bypassing some of this cycle by employing a glyoxylate shunt for, as yet, unclear reasons.

Together, these results indicate that strain ST4274, when cultivated on xylose, can utilize the glyoxylate shunt whilst concomitantly respiring using xylose, confirming this sugar as a non-fermentable carbon source. The up-regulation of hexokinase 1, glucokinase, fructose-1,6-bisphosphatase, trehalose-6-phosphate synthase, and acid trehalase, i.e. *HXK1*, *GLK1*, *FBP1*, *TSL1*, *TPS2*, and *ATH1* respectively, all indicate that cells have gluconeogenic activity [47]. A result that agrees with previous findings by Scalcinati et al. [43]. The up-regulation of phosphoglucomutase and UDP-glucose pyrophosphorylase, two isoenzymes of glycogen synthase and aquaglyceroporins *PGM2*, *UGP1*, *GSY1*, *GSY2* and *FPS1* also suggest that the cells are responding to xylose by accumulating storage carbohydrates, a starvation response phenotype. Indeed, this has been previously shown for slow growing respiring cells, indicative of cells utilising storage carbohydrates, such as trehalose and glycogen, to complete the cell cycle when nutrients are deprived [48–50]. This finding could explain why glucose transporters such as *HXT2*, *HXT5*, and *HXT6,* are up-regulated during growth on xylose, as this would ensure maximum uptake. This is despite these transporters, which can uptake both carbon sources, having a lower affinity for xylose (Fig. 3a) [51, 52].

With respect to the pentose phosphate pathway (PPP), RNA expression levels suggest a lower flux through the oxidative branch, in particular as the three key enzymes glucose-6-phosphate dehydrogenase, 6-phosphogluconolactonase and 6-phosphogluconate dehydrogenase, encoded by *ZWF1*, *SOL3*, and *GND1* respectively, had decreased expression compared to growth on glucose. On the other hand, transketolase *TKL2* in the non-oxidative branch of the PPP, showed a significant up-regulation compared with cells grown on glucose (Figs. 2a, b, 3) [53]. In contrast, the expression levels of transketolase *TKL1* and transaldolase *TAL1* were reduced compared with glucose conditions, suggesting the switch from glucose to xylose metabolism affects enzymes in the PPP differently (Fig. 3). However, overall it appears that the flux through the PPP decreases upon switching to xylose (Fig. 3).

**Fig. 4:**
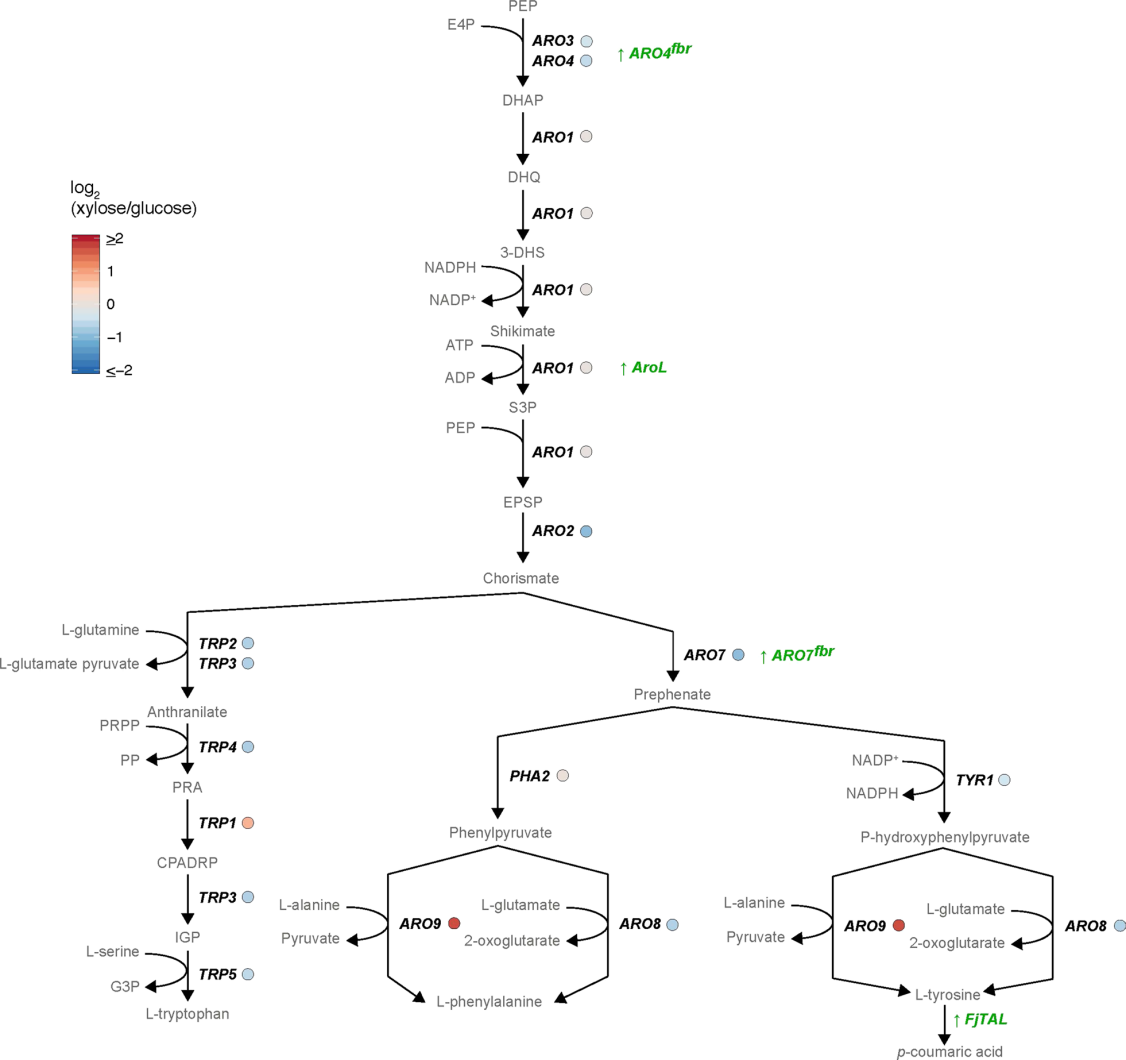
Gene expression levels of aromatic amino acid biosynthesis. The comparative analysis includes the log_2_ fold-change (log_2_FC) xylose/glucose under carbon limitation conditions. The green label indicates overexpressed enzymes, “fbr” indicates feedback-resistant

*p*-Coumaric acid is produced by *Saccharomyces cerevisiae* via the aromatic amino acid (AAA) “shikimate” pathway (Fig. 4). Here, phosphoenolpyruvate, PEP, and erythrose-4-phosphate, E4P, from glycolysis and the PPP respectively, are the primary substrates for two enzymes within the AAA pathway, specifically isoenzymes of DAHP synthase *ARO3* and *ARO4. DAHP* is converted to shikimate, SHIK, via 3-dehydroquinate, DHQ, and 3-dehydroshikimate, 3-DHS. 3-DHS is then converted to 5-enolpyruvylshikimate 3-phosphate EPSP, with all reactions being catalyzed by the Penta-functional enzyme, *ARO1*. EPSP is then converted by chorismate synthase *ARO2,* to chorismate, the precursor to all three aromatic amino acids, l-tryptophan, l-phenylalanine and l-tyrosine and subsequently to *p*-coumaric acid, from l-tyrosine. Interestingly, our RNA-seq data shows that for all reactions mentioned involving *ARO3, ARO4*, and *ARO2,* the related transcripts are down-regulated (Fig. 3). This result was unexpected as it contrasts with observations found via physiological characterisation, specifically the higher production levels of *p*-coumaric acid under xylose conditions (Table 2). One explanation for this could be that post-transcriptional regulation has an influence in this pathway. Nonetheless, this data suggests that xylose imposes strong regulatory effects on the expression level of the shikimate pathway, a result that matches GO term enrichment analysis, which found general amino acid biosynthesis to be down regulated on switching to xylose [54]. A result indicative of this pathway enhancing *p*-coumaric acid production via post-transcriptional regulation.

## Discussion

In this work, we report the first *S. cerevisiae* strain (ST4274) able to produce *p*-coumaric acid using xylose as the sole carbon source. Moreover, this strain achieved a 45-fold increase in titer of *p*-coumaric acid compared with production with glucose under our conditions (Table 2). Our physiological characterization is also consistent with transcriptome analysis of cells grown on xylose when focusing on central carbon metabolism (Fig. 3), with most significant GO-terms in the strain ST4274 being related to functions or features linked to respiratory processes, transport activities, biosynthetic processes and membrane functions (Fig. 2).

Physiological characterization under batch and chemostat conditions of the strain ST4274 presented low production rates of acetate, glycerol and ethanol compared to glucose condition (Table 2). The observed up-regulation of the glyoxylate pathway on xylose condition correlates with low dilution rates in carbon-limited chemostats in *S. cerevisiae* suggesting that this phenomenon is not limited to growth on xylose but is a common feature of respiratory metabolism (that occurs at low dilution rates with carbon limitation) [41, 46, 55].

On the one hand, ST4274 efficiently ferment glucose to produce ethanol by downregulating irrelevant metabolic pathways even though oxygen is present, this regulatory system is commonly defined as overflow metabolism or ‘Crabtree effect’. On the other hand, employing non-fermentable sugars, such as xylose, ST4274 showed a respiratory metabolism, and an up-regulation of non-fermentative pathways redirecting the carbon flux through AAAs biosynthesis leading a higher production of *p*-coumaric acid. An active respiratory metabolism, higher biomass yield, and a clearly diminished overflow metabolism lead to a low ethanol rate on xylose (~ 0.002 g/gDW h) compared to glucose (1.37 ± 0.38 g/gDW h). Recent studies on recombinant *S. cerevisiae* strain engineered for 3-HP production has shown a twofold change in the specific yield on xylose [56]. A similar observation was found in the production of amorphadiene, sesquiterpene molecule, in xylose cultures with *S. cerevisiae*. After a metabolic engineering approach, lead a higher titer and yield of amorphadiene on xylose (254.3 ± 6.2 mg/L, 6.25 ± 0.15 mg/g xylose) compared to glucose condition (120.2 ± 4.3 mg/L, 2.82 ± 0.10 mg/g) [57]. Co-cultures of xylose and glucose are essential for an efficient conversion of lignocellulose, still, up-to-date most strains exhibits glucose repression. To circumvent the glucose repression, co-cultures of xylose and cellobiose has been demonstrated to be feasible by Turner et al. with and engineered *S. cerevisiae* to produce value-added compounds from such as lactic acid reaching a titer of 83 g/L with a yield of 0.66 lactic acid/g sugar with low yields of ethanol [58]. A clearly diminished Crabtree effect and a respiratory metabolism in *S. cerevisiae* grown on xylose or co-cultures wit xylose and cellobiose have presented a low production of ethanol. However, the metabolism of *S. cerevisiae* on xylose has been proven to be advantageous to produce above described value-added compounds instead of ethanol.

To enhance *p*-coumaric acid production, we overexpressed the feedback-resistant versions of Aro4p and Aro7p, which can both be feedback regulated by l-tyrosine. These and other modifications subsequently led to a significant increase in *p*-coumaric levels (42-fold higher than previously reported with glucose, and the first report of *p*-coumaric acid production with xylose as the sole carbon source). Contrastingly, when analysing the transcriptome of xylose cultured cells, we found that many genes in the aromatic amino acid pathway, generating precursors for *p*-coumaric acid, were downregulated. This suggests that cells respond to these modifications by downregulating native genes involved in aromatic amino acid production, including *ARO4* and *ARO7*. A result, which suggests that aromatic amino acid levels are tightly regulated by the cell, at least at the transcriptional level, and that post-transcriptional regulation may predominate in this pathway.

## Conclusion

In conclusion, we designed a *p*-coumaric acid strain that presented a 45-fold increase compared to the same strain grown on glucose under the conditions we outline here. We also performed a transcriptome analysis to understand how the cell was able to re-wire its metabolism to enable *p*-coumaric acid production and found that cells produce this aromatic amino acid derivative by altering flux through central carbon metabolism, increasing storage carbohydrates, and in particular implementing respiratory metabolism.

## Materials and methods

### Strains and plasmids

All chemicals unless otherwise stated were purchased from Sigma-Aldrich (Merck). The *E. coli* strain DH5α was used as a host for plasmid propagation and standard cloning; cells were grown at 37 °C in Luria–Bertani (LB) medium containing 100 mg/L ampicillin or 50 mg/L kanamycin. DNA fragments were amplified by PCR using primers as described in Table 3. The biobricks are listed in Table 4. All engineered yeast strains and plasmids are described in Table 5, with all strains being constructed from CMB.GS010 *TRP1 ura3∆ his3∆ leu2∆,* originally derived from CEN.PK 113-7D [28]. The genetic modifications were performed by employing integrative EasyClone vectors with auxotrophic selection markers reported by Rodriguez [44]. The knockout strain *Δaro10Δpdc5* was constructed using a method based on a cloning-free, PCR-based allele replacement protocol by Erdeniz et al., which involved iterative replacement of these targeted genes with the cassettes KanMX and *LEU2* in the background strain ST2488 [59]. For *ARO10*, the knockout fragments BB828 and BB829 were transformed into *S. cerevisiae* and the transformants were selected in synthetic complete (SC) leucine drop-out medium (SC-Leu). For the *PDC5* knockout, the fragments BB1189 and BB1190 were transformed into the strain and the transformants were selected in SC medium supplemented with 200 mg/L G418 disulfate salt. Finally, the knockouts were confirmed by PCR on genomic DNA preparations, the *LEU2* marker was looped-out and the plasmids PL826, PL1964 and PL2747 were integrated into the strain.

**Table 3:** Primers used in this study

ID	Name	Sequence
Overexpression primers		
1691	Fj_TAL_1_fw	AGTGCAGGUAAAACAATGAACACCATCAACGAATATCTGAGC
1692	Fj_TAL_1_rv	CGTGCGAUTTAATTGTTAATCAGGTG
6785	Ec_aroL_2_fw	ATCTGTCAUAAAACAATGACACAACCTCTTTTTCTGA
6786	Ec_aroL_2_rv	CACGCGAUTCAACAATTGATCGTCTGTGC
1398	Sc_ARO7_1_fw	AGTGCAGGUAAAACAATGGATTTCACAAAACCAGAAAC
1399	Sc_ARO7_1_rv	CGTGCGAUTCACTCTTCCAACCTTCTTAGCAAG
1396	Sc_ARO4_2_fw	ATCTGTCAUAAAACAATGAGTGAATCTCCAATGTTCG
1397	Sc_ARO4_2_rv	CACGCGAUTCATTTCTTGTTAACTTCTCTTCTTTG
Verification primers		
904	Sc_X-3-out-seq_rv	CCGTGCAATACCAAAATCG
906	Sc_X-4-out-seq_rv	GACGGTACGTTGACCAGAG
912	XI-3- down-out_rv	CACATTGAGCGAATGAAACG
2220	Sc_ColoPCR_fw	CCTGCAGGACTAGTGCTGAG
1384	Sc_PDC5_Start_fw	AAAGCCTCCATATCCAAAG
1385	Sc_PDC5_End_rv	AGGTATGGTTAAAGATCACAC
1386	Sc_ARO10_Start_fw	ACCGAAATTTAAAAAAGCAG
1387	Sc_ARO10_End_rv	GTTTTCGGATAAAACTTCTTC
Knockout primers		
1368	Pdc5 _UP_fw	CGTAAACCTGCATTAAG
1369	Pdc5 _UP_rv	GATCCCCGGGAATTGCCATTGTGTTGTTCTCTTTG
1370	Pdc5_END_fw	GGTACCCAATTCGCCCTAGATTCAACGTTTGTGTA
1371	Pdc5_END_rv	CTAAGATCATAGCTAAAGG
1372	Aro10_UP_fw	GGATAGCCGTCATTTAC
1373	Aro10_UP_rv	GATCCCCGGGAATTGCCAGAGGGTTGATCAGTTAAA
1374	Aro10_END_fw	GGTACCCAATTCGCCCTACTACCAATTGTTCGTTT
1375	Aro10_END_rv	CGATAGGAATGACAGAA
476	KanMX_UP_fw	TGGCAATTCCCGGGGATCACGCTGC AGGTCGACAAC
477	KanMX_UP_rv	AGTGACGACTGAATCCGGTG
478	KanMX_END_fw	AATGGGCTCGCGATAATGTC
479	KanMX_END_rv	TAGGGCGAATTGGGTACCGCCACTAGTGGA TCTGATATCAC
150	LEU_UP_rv	CAGAAGCATAACTACCCATTCC
151	LEU_END_Fw	TGGAAGAGGCAAGCACGTTAGC
92	URA3_2/3_START_rv	CGCTTCCCATCCAGCATTTC
93	URA3_2/3_END_fw	CTGTCGTTCCATTGAAAGC

**Table 4:** Biobricks used in this study

BioBrick ID	Template for PCR	Fw primer	Rv primer
BB0380 (Fj_tal<-)	*F. johnsoniaeu* codon-optimized synthetic gene	Fj_Tal_U1_fw (ID1691)	Fj_TalU1_rv (ID1692)
BB254 (KlLEU2_2/3_START)	p0019(pUG73)	Sc_LEU2_2/3_START_fw (ID476)	Sc_LEU2_2/3_START_rv (ID150)
BB251	CEN.PK113-7D gDNA	Sc_Pdc5_UP_fw (ID1368)	Sc_Pdc5_UP_rv (ID1369)
BB252	CEN.PK113-7D gDNA	Sc_Pdc5_END_fw (ID1370)	Sc_Pdc5_END_fw (ID1371)
BB681	CEN.PK113-7D gDNA	Sc_Aro10_UP_fw (1372)	Sc_Aro10_UP_rv (1373)
BB0501 (Ec_AroL->)	EcoMG1655 ATCC 31884 gDNA	Ec_AroL_U2_fw (ID6785)	Ec_AroL_U2_rv (ID6786)
BB0361 (Sc_Aro7_G141S<-)	p0761 (pESC-URA-ARO7pm)	Sc_aro7_U1_fw (ID1398)	Sc_aro7_U1_rv (ID1399)
BB0364 (Sc_Aro4_K229L->)	p0775 (pESC-HIS-ARO4pm)	Sc_aro4_U2_fw (ID1396)	Sc_aro4_U2_rv (ID1397)
BB1189 (Pdc5_Up_kanmx_2/3 start)	p0015 (pUG6)	Pdc5_UP_fw (ID1368)	URA3_2/3_START_rv (ID92)
BB1190(kanmx_2/3_end_Pdc5_down)	p0015 (pUG6)	URA3_2/3_END_fw (ID93)	Pdc5_END_rv (ID1371)
BB828 (aro10_UP_Leu2_2/3_start)	BB251, BB245	Sc_Aro10_UP_fw (1372)	Sc-LEU2_2/3_START_rv (ID150)
BB829 (Leu2_2/3_end_aro10_down)	BB681, BB252	LEU2_2/3_END_fw (ID151)	Aro10_END_rv (ID1375)

**Table 5:** Plasmids and strains used in this study

Name	Parental plasmids	Description	Reference
Plasmids			
pCfB258		Integrative plasmid, pX-4-loxP, SpHIS5, P_*TEF1*_-T_*ADH1*_, P_*GKp1*_-T_*CYC1*_	[21]
pCfB390		Integrative plasmid, pXI-3-loxP, *KlURA3*, P_*TEF1*_-T_*ADH1*_, P_*PGK1*_-T_*CYC1*_	[21] ]
pCfB258		Integrative plasmid, pX-4-loxP, *SpHIS5*, P_*TEF1*_-T_*ADH1*_, P_*PGK1*_-T_*CYC1*_	[21]
pCfB0826		Integrative plasmid, pX-4-LoxP, *SpHiS5,* P_*TEF1*_-*ScAro7*^fbr^-T_*ADH1*_, P_*PGK1*_-*ScAro4*^fbr^-T_*CYC1*_	[44]
pCfB1964		Integrative plasmid, pX-2-loxP, KlURA3, P_TEF1_-*Fj_TAL*-T_ADH1_	[44]
pCfB2747	pCfB3034	Integrative plasmid, X-3, P_PGK1_- *EcaroL*, *KlLEU2*	[44]
pCfB3524	pCfB390	Integrative plasmid, pXI-3-loxP, *KlURA3*, BB380 (*Fj*_*TAL*<-), BB010 (<-P_*TEF1*_-P_*PGK1*_->), BB0501 (*EcaroL*->)	This study

Name	Description	Reference	
Strains			
ST2488	CEN.PK 113-3C/pRS314-X123 (P_TDH3_-*PsXYL1*, P_TDH3_-*PsXYL2*, P_TDH3_-*PsXYL3 TRP1*) *TRP1 ura3Δ his3Δ leu2Δ*	[56]	
ST4274	CMB.GS010Δaro10::Leu27/pRS314-X123 (P_TDH3_-*PsXYL1*, P_TDH3_-*PsXYL2*, P_TDH3_-*PsXYL3 TRP1*) Δ*pdc5*::Kanmx *TAL AroL*::*URA3 Aro7*-*Aro4*::*His5*	This study	

### Media and growth conditions

All yeast strains were cultivated at 30 °C using 20 g/L of either glucose or xylose as the carbon source in rich medium (YP, 1% (w/v); Bacto yeast extract, 2% (w/v) Bacto peptone), SC medium, or SC drop-out media (SC-Ura, SC-Leu, SC-His) with agar plates prepared using pre-mixed drop-out powder, supplemented with 20 g/L agar. Minimal mineral (MM) medium for all fermentations was prepared as described previously [60], containing per litre: 25 g xylose or 25 g of glucose, 5 g (NH_4_)_2_SO_4_, 3 g KH_2_PO_4_, 0.5 g MgSO_4_·7H2O, 0.1 mL antifoam 204 (Sigma A-8311), as well as 1 mL vitamin solution and 1 mL trace metal solution prepared as described previously [2]. The medium used for preparing inoculums in shake-flasks was the same as that for fermentations, with the following modifications: no antifoam, 7.5 g/L (NH_4_)_2_SO_4_, 14.4 g/L KH_2_PO_4_ and the pH was adjusted to 6 with KOH.

### Bioreactor cultivations

All fermentations (batch and chemostat) were performed in controlled conditions and conducted in MM medium. A single colony of ST4274 was inoculated into 25 mL of MM medium supplemented with 2% of Glucose or Xylose as above, and incubated in 250-mL baffled shake flasks at 30 °C with shaking 250 rpm for 18 h. Optical density OD_600_ was measured during the exponential phase and an appropriate volume of inoculum was spun down at 4000×*g* for 10 min at 4 °C. Then the pellet was resuspended in 10 mL of fresh MM and used to inoculate the bioreactor with an initial OD_600_ of 0.05. The batch and chemostat fermentations were performed in 1 L DASGIP Bioreactors (Dasgip, Jülich, Germany), with the following conditions for batch fermentation: working volume: 0.6 L, temperature set-point: 30 °C, airflow: 1 vvm (gas volume flow per unit of liquid volume per minute), pH: 6 (via feedback-controlled addition of 2 M KOH), dissolved oxygen: 30% (via feedback control of agitation from 600 rpm to a maximum of 1200 rpm). The concentration of O_2_ and CO_2_ in the exhaust gas was monitored by a DASGIP^®^ GA4 exhaust analyser. Chemostat fermentations were preceded by a batch fermentation, under the same conditions of temperature, pH and stirring conditions as previously mentioned. After glucose and xylose had been depleted by a drop in the CO_2_ production rate, the fermentations were switched to chemostat mode yielding a dilution rate (*D*) of 0.05 h^−1^ with a feeding solution of 7.5 g/L of glucose and 15 g/L of xylose. The working volume was kept constant at 0.6 L with an overflow system that continuously removed excess medium. To ensure cells were growing at a steady-state, chemostats were run for at least four residence times before sampling at a dilution rate of 0.05 h^−1^. Here, after 80 h of chemostat cultivation, samples were taken for cell dry weight (CDW), *p*-coumaric acid and organic acid quantification as well as RNA sequencing. CDW was determined in duplicate by filtering 15 mL of cell broth through dried, pre-weighed 0.45 μm polyethersulfone (PES) membranes (Sartorius Stedim, Aubagne, France) and washing with deionized water. Membranes were then dried in a microwave oven at 120 W for 15 min and placed in a silica gel desiccator for a minimum of 12 h before re-weighing.

### Quantification of sugars, extracellular metabolites and *p*-coumaric acid

Samples withdrawn from bioreactor cultures were filtered using 0.2 μm nylon filters then immediately stored at − 20 °C before analysis. Glucose, xylose and extracellular metabolites (acetate, ethanol, glycerol, pyruvate, and succinate) were analyzed using high-performance liquid chromatography (HPLC; Ultimate 3000, Dionex, Sunnyvale, CA, USA) using an Aminex HPX87-H column (Bio-Rad Laboratories, Munich, Germany). Mobile phase (eluent) used was 5 mM H_2_SO_4_ (0.6 mL/min), with the column temperature maintained at 45 °C. Ethanol, glucose, and glycerol were analyzed using a refractive index detector (Shodex RI-101, Showa Denko, New York, NY, USA), while acetate was analyzed using the UV detector set at 210 nm. Six-point standard curves were set for quantification. Evaporation of ethanol was compensated for by assuming an evaporation rate of 0.0068 mmol_ethanol evaporated_ ${\text{mmol}}_{{{\text{ethanol in solution}}^{ - 1} {\text{h}}^{ - 1} }}$, of the ethanol present at each specific time point [61].

In order to quantify *p*-coumaric acid, samples were withdrawn from the bioreactor during the time course cultivation (1 mL each) then diluted 1:1 with absolute ethanol (100% v/v). After dilution, samples were spun down at max speed (10,000 rpm). Quantification of *p*-coumaric acid was performed on HPLC (Thermo), equipped with a Discovery HS F5 150 mm × 2.1 mm column (particle size 3 mm) according to Rodriguez et al. and the area under the peak was integrated with Chromeleon 7 and used for quantification by fitting with a standard curve [44].

### RNA sequencing

Concentration and quality of the nucleic acids prior to sequencing were determined using a Qubit 2.0 fluorometer (Invitrogen) and an Agilent 2100 Bioanalyzer (Agilent Technologies) respectively. Sequencing libraries were prepared in triplicates using a TruSeq stranded mRNA Sample Preparation kit (Illumina Inc., San Diego CA) and were pooled together before sequencing. An average cDNA library size was determined using the Agilent DNA 1000 kit on an Agilent 2100 Bioanalyzer. Normalized libraries were combined in 10 mM Tris–Cl at pH 8, tween 20 (0.05%) to a final concentration of 10 nM, then libraries were denatured in 0.2 N NaOH. A pool of 1.3 pM of each library was resuspended in 1.3 mL ice-cold HT1 buffer, then loaded onto the flow cell provided in the NextSeq 500/550 High Output kit v2 (300 cycles) and sequenced on a NextSeq^®^ (Illumina Inc., San Diego CA) platform with a paired-end protocol and read lengths of 151 nt. Reads were aligned on the yeast genome by using Bowtie2 [62] and further processed by SAMTools [51] and BEDTools [63] to count the number of reads aligning to each gene. Differential gene expression was analyzed using the DEseq package in R programming language [64].

### Differential mRNA expression

Differential expression was determined for strain ST4274 using fold change in transcript abundance when cells were grown on xylose relative to the glucose reference condition. The p values were adjusted for multiple testing using Benjamini–Hochberg procedure [65] as implemented in DESeq. Threshold values for differentially expressed mRNAs were adjusted to p < 0.01 and a > 0.5, < − 0.5 log_2_ fold change (FC) for both up and down regulated genes was applied. A list of differentially expressed genes were annotated with gene ontology (GO) terms (Process, Function and Component) using the Bioconductor R package BioMaRt to access the Ensembl *Saccharomyces cerevisiae* gene data set and the list of the GO names (name 1006) for each ensemble gene ID. Gene set analysis and network-based plots of overlapping gene sets and their significance were generated with the Bioconductor R package piano [66]. Volcano plots for visualising log_2_ FC versus their significance was performed using the R package ggplot2 [67].

## Data Availability

The generated RNAseq and fermentations datasets from this current study are available from the corresponding author on reasonable request.
